# Lignin Engineering in Forest Trees

**DOI:** 10.3389/fpls.2019.00912

**Published:** 2019-07-25

**Authors:** Alexandra Chanoca, Lisanne de Vries, Wout Boerjan

**Affiliations:** ^1^Department of Plant Biotechnology and Bioinformatics, Ghent University, Ghent, Belgium; ^2^VIB Center for Plant Systems Biology, Ghent, Belgium

**Keywords:** lignin, forest trees, genetic engineering, CRISPR, field trial

## Abstract

Wood is a renewable resource that is mainly composed of lignin and cell wall polysaccharides. The polysaccharide fraction is valuable as it can be converted into pulp and paper, or into fermentable sugars. On the other hand, the lignin fraction is increasingly being considered a valuable source of aromatic building blocks for the chemical industry. The presence of lignin in wood is one of the major recalcitrance factors in woody biomass processing, necessitating the need for harsh chemical treatments to degrade and extract it prior to the valorization of the cell wall polysaccharides, cellulose and hemicellulose. Over the past years, large research efforts have been devoted to engineering lignin amount and composition to reduce biomass recalcitrance toward chemical processing. We review the efforts made in forest trees, and compare results from greenhouse and field trials. Furthermore, we address the value and potential of CRISPR-based gene editing in lignin engineering and its integration in tree breeding programs.

## Introduction

Fossil resources are the main feedstock for energy and organic compounds, and their use results in the emission of greenhouse gases associated with climate change. The coming climate crash calls for an urgent transition from a fossil-based to a bio-based economy in which lignocellulosic biomass rather than oil is used for the production of fuels, chemicals and materials. Wood is an important source of lignocellulosic biomass; it is mainly composed of secondary-thickened cell walls rich in cellulose, hemicelluloses, and lignin. All three polymers can be valorized in the bio-based economy. Cellulose is a source for the pulp and paper industry, and both cellulose and hemicelluloses can be depolymerized to their monosaccharides for fermentation into, e.g., bio-ethanol, lactic acid and detergents (Vanholme et al., 2013b). As lignin negatively affects the efficiency of wood processing toward these applications, trees can be engineered to accumulate less lignin, to become more amenable for the production of paper and fermentable sugars. On the other hand, lignin is increasingly being considered a valuable component in the bio-based economy. Indeed, given that lignin is the largest renewable aromatic source on Earth, the economic viability of a bio-refinery can be significantly increased if lignin is also valorized, and used as a resource for the production of chemicals (Holladay et al., 2007; Tuck et al., 2012; Davis et al., 2013; Ragauskas et al., 2014; Li C. et al., 2015; Van den Bosch et al., 2015; Rinaldi et al., 2016; Upton and Kasko, 2016; Schutyser et al., 2018).

**FIGURE 1:**
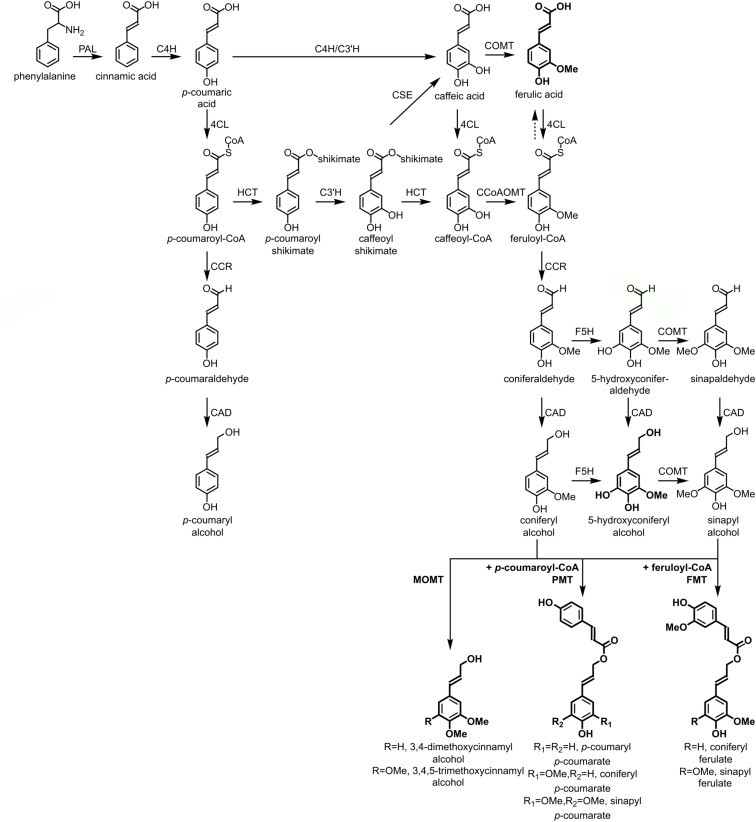
Lignin biosynthetic pathway. Alternative monomers and heterologously expressed enzymes are shown in bold. PAL, phenylalanine ammonia-lyase; C4H, cinnamate 4-hydroxylase; 4CL, 4-coumarate:CoA ligase; HCT, *p*-Hydroxycinnamoyl-CoA:quinate/shikimate-*p*-hydroxycinnamoyltransferase; C3’H, *p*-coumaroyl quinate/shikimate 3’-hydroxylase; CSE, caffeoyl shikimate esterase; CCoAOMT, caffeoyl-CoA *O*-methyltransferase; CCR, cinnamoyl-CoA reductase; F5H, ferulate 5-hydroxylase/CAld5H, coniferaldehyde 5-hydroxylase; COMT, caffeic acid *O*-methyltransferase; CAD, cinnamyl alcohol dehydrogenase; FMT, feruloyl-CoA monolignol transferase; PMT, *p*-coumaroyl-CoA monolignol transferase.

The lignin polymer is composed of monolignols that are produced by the phenylpropanoid and monolignol biosynthetic pathways, by a series of enzymatic reactions starting with the deamination of phenylalanine (Figure 1). The monolignols are synthesized in the cytoplasm and translocated to the apoplast, where they are dehydrogenated to monolignol radicals by the action of laccases and peroxidases (Berthet et al., 2011; Zhao et al., 2013). These monolignol radicals then couple with each other in a combinatorial fashion, generating a range of chemical bonds such as the aryl-ether bond (β-O-4), resinol bond (β-β), and phenylcoumaran bond (β-5) (Boerjan et al., 2003; Ralph et al., 2004; Vanholme et al., 2010). The most common monolignols are the hydroxycinnamyl alcohols *p*-coumaryl, coniferyl, and sinapyl alcohols, which generate the H, G, and S units upon their incorporation into the lignin polymer, respectively (Bonawitz and Chapple, 2010; Ralph et al., 2019; Vanholme et al., 2019). The relative contribution of the lignin building blocks varies among taxa, developmental stage, tissue and cell type, and even cell wall layer; lignin from softwoods (gymnosperms) is comprised almost entirely of G units with a minor fraction of H units, while lignin from hardwoods (angiosperms) has S units in addition to G units and traces of H units (Boerjan et al., 2003; Vanholme et al., 2010, 2019). Besides these traditional monolignols, a variety of other *p*-hydroxylated aromatic molecules can be incorporated in the lignin polymer to various levels (Vanholme et al., 2019).

Given that lignin is a major recalcitrance factor in wood delignification processes, large research efforts have been devoted to unravel the lignin biosynthetic pathway, and to study the effects of perturbations of the lignin biosynthesis genes on lignin amount and composition, and on wood processing efficiency. While modifications in genes ranging from those encoding transcription factors up to those encoding oxidative enzymes have resulted in altered lignin content, composition or deposition (Eriksson et al., 2000; Li Y.H. et al., 2003; Liang et al., 2008; Lu et al., 2013; Lin et al., 2016; Xu et al., 2017; Yang et al., 2017; Obudulu et al., 2018), this review will focus on the results obtained by engineering the lignin biosynthetic genes.

## Engineering the Lignin Pathway

Table 1 provides an overview of the different studies on downregulated or mutated lignin biosynthetic genes in poplar, pine, eucalyptus and birch, with the resulting effects on wood processing efficiencies, when determined. Reducing the activity of any step of the lignin biosynthetic pathway, starting from PAL up to CAD may result in a reduction in lignin content (Table 1). Several parameters influence the degree of lignin reduction, such as the target gene and the degree of downregulation of the enzyme activity, which in turn depends on the efficiency of the silencing construct used, the size of the gene family, and redundancy within the gene family. Generally, the downregulation of the steps from *C4H* up to *CCR* results in a more dramatic reduction in lignin amount (Hu et al., 1999; Meyermans et al., 2000; Zhong et al., 2000; Li L. et al., 2003; Jia et al., 2004; Lu et al., 2004; Leplé et al., 2007; Coleman et al., 2008a,b; Bjurhager et al., 2010; Mansfield et al., 2012; Ralph et al., 2012; Min et al., 2014; Van Acker et al., 2014; Zhou et al., 2015, 2018; Saleme et al., 2017; Xiang et al., 2017) than downregulation of *F5H*, *COMT* and *CAD* (Van Doorsselaere et al., 1995; Baucher et al., 1996; Lapierre et al., 1999; Jouanin et al., 2000; Van Acker et al., 2014; Wang et al., 2018). Lignin reduction can be associated with an increase in S/G, such as in *C3’H*- (Coleman et al., 2008a; Ralph et al., 2012) and *CCoAOMT*-downregulated trees (Meyermans et al., 2000), or a decrease in S/G ratio such as in *CSE-* (Saleme et al., 2017), and *COMT-*downregulated trees (Van Doorsselaere et al., 1995; Lapierre et al., 1999; Jouanin et al., 2000). Interestingly, low-lignin *4CL*-downregulated poplars were found to have an increase in S/G (Min et al., 2014; Xiang et al., 2017), a decrease in S/G (Voelker et al., 2010; Zhou et al., 2015), or ratios comparable to wild type (Hu et al., 1999; Li L. et al., 2003). This variance cannot be associated with the promoter or the method used for downregulation, suggesting that differences in the degree of silencing, growth conditions or developmental state influence this trait. On the other hand, the strongest effects on H/G/S lignin composition have been observed for trees downregulated in *C3’H* and *HCT*, which deposit lignin with large increases in H unit content (Coleman et al., 2008a; Ralph et al., 2012; Vanholme et al., 2013a), whereas trees that overexpress *F5H* produce lignin strongly enriched in S units (Franke et al., 2000; Li L. et al., 2003; Stewart et al., 2009), and trees that are downregulated in *COMT* have dramatically reduced S unit biosynthesis (Van Doorsselaere et al., 1995; Lapierre et al., 1999).

**TABLE 1:** Overview of forest trees with modified expression of lignin biosynthesis genes.

**Species**	**Gene**	**Method**	**Growth conditions**	**Lignin amount**	**Lignin composition**	**Saccharification efficiency**	**Pulping efficiency**	**Biomass yield**	**References**
*Pinus radiata*	*HCT*	RNAi	TE cultures	↓42%	↑ H/G	n.d.	n.d.	n/a	Wagner et al., 2007
*Pinus radiata*	*CCoAOMT*	RNAi	TE cultures	↓20%	↑ H/G	n.d.	n.d.	n/a	Wagner et al., 2011
*Pinus radiata*	*CCR*	RNAi	TE cultures	↓46%	Trace amount of ferulic acid	n.d.	n.d.	n/a	Wagner et al., 2013
*Pinus radiata*	*F5H + COMT*	Heterologous (over) expression	TE cultures	n.d.	Incorporation of S units	n.d.	n.d.	n/a	Wagner et al., 2015
*Betula pendula*	*COMT*	Co-suppression	Greenhouse	WT	↓ S/G	n.d.	n.d.	WT/↓	Tiimonen et al., 2005
*Leucaena leucocephala*	*COMT*	Antisense	Greenhouse	↓30%	↓ S units (histochemical)	n.d.	↑	WT	Rastogi and Dwivedi, 2006
*Pinus radiata*	*4CL*	RNAi	Greenhouse	↓36%	↑ H/G	n.d.	n.d.	↓	Wagner et al., 2009
*Pinus taeda*	*4CL*	Xylem-specific RNAi	Greenhouse	↓33%	n.d.	↑	n.d.	n.d.	Edmunds et al., 2017
*Pinus taeda*	*CAD*	Mutant allele	Greenhouse	n.d.	↑ cinnamaldehydes, ↑ benzaldehyde, ↑ dihydroconiferyl alcohol, ↓ G units	n.d.	n.d.	n.d.	Ralph et al., 1997
				↓9%	↑ coniferaldehyde	n.d.	n.d.	WT	MacKay et al., 1997
				n.d.	↑ dihydroconiferyl alcohol, ↑ vanillin, ↑ coniferaldehyde, ↑ H/G	n.d.	n.d.	n.d.	Lapierre et al., 2000
*Pinus taeda*	*F5H + COMT + SAD/CAD*	Heterologous (over) expression	Greenhouse	WT	Incorporation of S units	WT	n.d.	WT	Edmunds et al., 2017
*Picea abies*	*CCR*	Antisense	Greenhouse	↓8%	↓ H/G	n.d.	↑	↓ diameter	Wadenbäck et al., 2008
*Eucalyptus urophylla × E. grandis*	*C3’H*	Antisense	Greenhouse	↓27%	↓ S/G, ↑ H units	↑	n.d.	↓ height	Sykes et al., 2015
*Eucalyptus urophylla × E. grandis*	*C4H*	Antisense	Greenhouse	↓36%	↓ S/G	↑	n.d.	↓ height	Sykes et al., 2015
*P. tremula × P.tremuloides*	*C4H*	Antisense	Greenhouse	↓33%	WT	n.d.	n.d.	↓ height	Bjurhager et al., 2010
*P. tremuloides Michx.*	*4CL*	Antisense	Greenhouse	↓45%	WT S/G	n.d.	n.d.	↑	Hu et al., 1999^*^
*P. tremuloides*	*4CL*	Downregulation	Greenhouse	Up to ↓40%	WT S/G	n.d.	n.d.	WT	Li L. et al., 2003^*^
	*4CL + F5H*	Downregulation + overexpression	Greenhouse	↓52%	↑ S/G	n.d.	n.d.	WT	
*P. tremuloides*	*4CL*	Antisense	Greenhouse	↓40%	WT S/G	n.d.	n.d.	WT	Li L. et al., 2003
*P. tomentosa*	*4CL*	Antisense	Greenhouse	↓42%	n.d.	n.d.	n.d.	WT	Jia et al., 2004
*P. trichocarpa*	*4CL*	Downregulation	Greenhouse	↓30%	WT S/V	↑	n.d.	n.d.	Min et al., 2012
*P. nigra L. × P. maximowiczii*	*4CL*	Antisense	Greenhouse	Up to ↓55%	↓ S/V/↑ S/V	n.d.	n.d.	n.d.	Min et al., 2014
*P. nigra L. × P. maximowiczii*	*4CL + F5H*	Antisense + antisense	Greenhouse	WT/↓	WT	n.d.	n.d.	n.d.	
*P. nigra L. × P. maximowiczii*	*4CL + F5H*	Antisense + overexpression	Greenhouse	↓	↑S/V	n.d.	n.d.	n.d.	
*P. tremula × P. alba*	*4CL1*	CRISPR/Cas9 mutants	Greenhouse	↓23%	↓S/G	n.d.	n.d.	WT	Zhou et al., 2015
*P. tremula × P. alba*	*4CL2*	CRISPR/Cas9 mutants	Greenhouse	WT	WT S/G	n.d.	n.d.	WT	Zhou et al., 2015
*P. nigra L. × P. maximowiczii*	*4CL*	Antisense	Greenhouse	↓47%	↑S/V	n.d.	n.d.	n.d.	Xiang et al., 2017
*P. alba × P. grandidentata*	*C3’H*	RNAi	Greenhouse	↓56%	↑H units, ↑S/G	n.d.	n.d.	n.d.	Coleman et al., 2008a
				n.d.	n.d.	n.d.	n.d.	↓	Coleman et al., 2008b
				WT/↓	n.d.	↑	↑	↓	Mansfield et al., 2012
*P. alba × P. grandidentata*	*C3’H*	Hairpin	Greenhouse	↓50%	↑H units, ↑ S/G	n.d.	n.d.	n.d.	Ralph et al., 2012
*P. alba × P. glandulosa*	*C3’H*	Downregulation	Greenhouse	↓30%	n.d.	n.d.	n.d.	↓ diameter	Zhou et al., 2018
*P. nigra*	*HCT*	Mutant allele	Greenhouse	WT	↑H units, ↑S/G	n.d.	n.d.	n.d.	Vanholme et al., 2013a
*P. alba × P. glandulosa*	*HCT*	Downregulation	Greenhouse	↓20%	n.d.	n.d.	n.d.	↓ diameter	Zhou et al., 2018
*P. tremula × P. alba*	*CSE*	Hairpin	Greenhouse	up to ↓25%	↑H units, ↓S/G	↑	n.d.	WT	Saleme et al., 2017
*P. tremula × P. alba*	*CCoAOMT*	Sense	Greenhouse	↓12%	↑ S/G, incorporation of *p*-hydroxybenzoic acid	n.d.	n.d.	WT	Meyermans et al., 2000
*P. tremula × P. alba*	*CCoAOMT*	Antisense	Greenhouse	Up to ↓40%	WT, incorporation of *p*-hydroxybenzoic acid	n.d.	n.d.	WT	Zhong et al., 2000
*P. tormentosa*	*CCoAOMT*	Antisense	Greenhouse	Up to ↓26%	n.d.	n.d.	n.d.	WT	Lu et al., 2004
*P. tremula × P. alba*	*CCR*	Antisense and co-suppression	Greenhouse	up to ↓30%	↓S/G, ferulic acid incorporation	n.d.	n.d.	WT	Leplé et al., 2007^*^
				↓19%	WT/↓	↑	n.d.	n.d.	Van Acker et al., 2014
*P. nigra L. × P. maximowiczii*	*F5H*	Antisense	Greenhouse	WT/↑	↓S/G	n.d.	n.d.	n.d.	Min et al., 2014
*P. tremuloides*	*F5H*	Heterologous OE	Greenhouse	WT	↑S/G	n.d.	n.d.	WT	Li L. et al., 2003
*P. tremula × P. alba*	*F5H*	Heterologous OE	Greenhouse	n.d.	↑ S/G	n.d.	n.d.	n.d.	Franke et al., 2000
				WT	↑ S/G	n.d.	↑	WT	Huntley et al., 2003
				↓	↑ S/G, ↓ *p*-hydroxybenzoic acid	n.d.	n.d.	WT	Stewart et al., 2009
				WT	↑ S/G	WT	↑	WT	Mansfield et al., 2012
*P. tremula × P. alba*	*COMT*	Antisense	Greenhouse	WT	↓ S/G, incorporation of 5-OH-G	n.d.	n.d.	WT	Van Doorsselaere et al., 1995^*^
				WT	↓ S/G	n.d.	↓	WT	Lapierre et al., 1999
*P. tremula × P. alba*	*COMT*	Co-suppression	Greenhouse	↓17%	↓ S/G, incorporation of 5-OH-G	n.d.	↑	WT	Jouanin et al., 2000
*P. tremula × P. alba*	*CAD*	Antisense and co-suppression	Greenhouse	WT	WT S/G, ↑ conjugated aldehyde moieties	n.d.	↑	WT	Baucher et al., 1996^*^
				↓	WT S/G	n.d.	↑	WT	Lapierre et al., 1999
*P. tremula × P. alba*	*CAD*	Hairpin	Greenhouse	↓10%	↓ canonical S/G, ↑ sinapaldehyde	↑	n.d.	WT	Van Acker et al., 2017
*P. tremula × P. alba*	*MOMT4*	Heterologous OE	Greenhouse	↓15%	↓ S/G, ↓ *p*-hydroxybenzoic acid	↑	n.d.	WT	Cai et al., 2016
*P. alba × P. grandidentata*	*PMT*	Heterologous OE	Greenhouse	WT	WT S/G, incorporation of *p*-coumarate conjugates	n.d.	n.d.	WT	Smith et al., 2015
*P. alba × P. grandidentata*	*FMT*	Heterologous OE	Greenhouse	WT	↑ S/G, incorporation of acylated monolignols	↑	n.d.	WT	Wilkerson et al., 2014
				n.d.	n.d.	↑	n.d.	n.d.	Kim et al., 2017
				WT	WT S/G	↑	n.d.	n.d.	Bhalla et al., 2018
*P. tremula × P. alba*	*4CL*	Antisense	Field trial	WT	Low S/G in brown wood	WT	n.d.	↓	Voelker et al., 2010
*P. tomentosa Carr.*	*4CL*	Sense	Field trial	Up to ↓30%	↑ S/G	n.d.	n.d.	↑	Tian X.M. et al., 2013
*P. nigra L. × P. maximowiczii A*	*4CL*	Antisense	Field trial	WT/ ↓	WT	↑	n.d.	n.d.	Xiang et al., 2017
*P. trichocarpa*	*4CL*	Antisense	Field trial	↓	↓ S/G	n.d.	n.d.	↓	Stout et al., 2014
*P. tomentosa*	*4CL*	Antisense	Field trial	Up to ↓10%	n.d.	WT	n.d.	WT	Wang et al., 2012
*P. tomentosa*	*CCoAOMT*	Antisense	Field trial	↓6-10%	n.d.	↑	n.d.	WT	Wang et al., 2012
*P. tremula × P. alba*	*CCoAOMT*	Antisense	Field trial	↓13%	Slight increment in S/G	n.d.	↑	WT	Wei et al., 2008
*P. tremula × P. alba*	*CCR*	Antisense and co-suppression	Field trial	Up to ↓47%	↓ S/G, Incorporation of ferulic acid	n.d.	↑	↓	Leplé et al., 2007
				Up to ↓24%	Incorporation of ferulic acid	↑	n.d.	↓	Van Acker et al., 2014
*P. tremula × P. alba*	*COMT*	Antisense	Field trial	WT	↓ S/G, incorporation of 5-OH-G	n.d.	↓	WT	Lapierre et al., 1999^*^
*P. tremula × P. alba*	*COMT*	Antisense	Field trial	WT	↓ S/G, incorporation of 5-OH-G	n.d.	↓	WT	Pilate et al., 2002^*^
*P. tremula × P. alba*	*CAD*	Sense and antisense	Field trial	Slightly ↓	WT S/G, ↑ free phenolic units	n.d.	↑	WT	Lapierre et al., 1999^*^
*P. tremula × P. alba*	*CAD*	Antisense	Field trial	Slightly ↓	WT S/G, ↑ free phenolic units	n.d.	↑	WT	Pilate et al., 2002^*^

n.d., not determined; n/a, not applicable; S/G, syringyl/guaiacyl ratio; S/V, syringaldehyde/vanillin ratio; H/G, p-hydroxyphenyl/guaiacyl ratio. Papers reporting plants that have been used for independent studies reporting on biotic or abiotic stress tolerance are shown with a ^*^. For abbreviations of gene names (see legend Figure 1). Lignin amount was determined by various methods, see the corresponding reference for specific information. Readers are referred to Wang et al. (2018) for additional raw data on downregulated lines for monolignol biosynthesis genes in P. trichocarpa.

Both the reduced lignin content and variation in the H/G/S ratios can affect the biomass processing efficiency. Consistent with the established role of lignin in determining biomass recalcitrance (Zeng et al., 2014; McCann and Carpita, 2015; Li et al., 2016; Wang et al., 2018), plants with reduced levels of lignin show increased chemical pulping and saccharification efficiency (Hu et al., 1999; Jouanin et al., 2000; Rastogi and Dwivedi, 2006; Wadenbäck et al., 2008; Wang et al., 2012; Sykes et al., 2015; Cai et al., 2016; Edmunds et al., 2017; Saleme et al., 2017; Xiang et al., 2017; Van Acker et al., 2017; Wang et al., 2018). An increased level of H units reduces lignin polymer length and, hence, increases the removal of lignin from the biomass (Mansfield et al., 2012; Sykes et al., 2015). Increased S/G results in lignin more easily cleaved and extracted in alkaline conditions, supposedly due to the lower degree of polymerization (Huntley et al., 2003; Stewart et al., 2009; Mansfield et al., 2012; Yoo et al., 2018).

The processing efficiency of the biomass can also be modified by the increased incorporation of molecules that generally represent minor components in the lignin of wild-type plants. The incorporation of ferulic acid in CCR-deficient trees results in the formation of acetal bonds in the lignin polymer, which are easily cleaved in acidic biomass pretreatments (Leplé et al., 2007; Ralph et al., 2008; Van Acker et al., 2014). Indeed, the levels of ferulic acid in lignin positively correlated with a higher saccharification efficiency (Van Acker et al., 2014). The incorporation of 5-hydroxyconiferyl alcohol and 5-hydroxyconiferaldehyde in the lignin of COMT-deficient poplars (Van Doorsselaere et al., 1995; Lapierre et al., 1999; Jouanin et al., 2000; Morreel et al., 2004; Lu et al., 2010) gives rise to benzodioxane bonds, potentially preventing covalent linkages between lignin and the polysaccharide hydroxyl groups (Weng et al., 2010; Vanholme et al., 2012; Nishimura et al., 2018). On the other hand, COMT deficiency also results in a more condensed lignin due to the relatively higher levels of the condensed β-β and β-5 bonds, and the lower levels of β-O-4 bonds, when the S unit frequency drops. Chemical pulping of wood derived from poplars strongly downregulated for COMT resulted in a higher pulp yield, counterbalanced by the residual lignin content in the pulp. These trees had a lower lignin and a higher cellulose content (Jouanin et al., 2000). On the other hand, poplars that were modestly downregulated for COMT had a large decrease in pulp yield, presumably because lignin content had remained normal while the lignin had a higher frequency of condensed bonds that negatively affected the lignin extraction (Lapierre et al., 1999; Pilate et al., 2002). The incorporation of cinnamaldehydes in the lignin polymer in CAD-deficient trees results in shorter lignin polymer chains, hence a higher proportion of free phenolic end groups that increase the solubility of the polymer in alkali. The incorporation of cinnamaldehydes in the lignin polymer presumably also reduces the covalent interaction of the aliphatic chain with hemicellulose, again rendering the lignin more soluble. In addition, due to the extended conjugated system that is generated when a cinnamaldehyde β-O-4 couples with another monomer, the aromatic ether bond of the incorporated cinnamaldehyde becomes more susceptible to alkaline cleavage (Lapierre et al., 1989; Van Acker et al., 2017).

Lignin polymerization is a combinatorial radical coupling process, allowing a wide range of phenolic compounds to be naturally incorporated into the lignin polymer (Boerjan et al., 2003; Vanholme et al., 2019). Researchers have attempted to tailor the lignin amount and composition to improve biomass processing by expression of heterologous genes, aiming at the biosynthesis and incorporation of various compatible phenolic compounds as alternative monolignols into the lignin polymer (Ralph, 2006; Vanholme et al., 2012; Mottiar et al., 2016; Mahon and Mansfield, 2018). One example is the introduction of genes encoding enzymes that are needed for S unit biosynthesis in pine; the simultaneous expression of *F5H*, *COMT* and *CAD* successfully introduced S units in *Pinus radiata* (Wagner et al., 2015; Edmunds et al., 2017). The introduction of the gene encoding a monolignol 4-O-methyltransferase (*MOMT4*) into poplar leads to the formation of 4-O-methylated coniferyl and sinapyl alcohols, which cannot be incorporated into the growing lignin polymer because they lack the aromatic hydroxyl group. This leads to a halt in lignin polymerization and results in trees with lower lignin content and higher saccharification efficiency (Bhuiya and Liu, 2010; Cai et al., 2016). Poplars have also been engineered to contain ester linkages in the lignin polymer backbone. Coniferyl ferulate esters were introduced into the polymer via expression of a *FERULOYL-CoA:MONOLIGNOL TRANSFERASE* (*FMT*) gene derived from *Angelica sinensis* (Wilkerson et al., 2014), leading to an improved saccharification efficiency under various pretreatment conditions (Wilkerson et al., 2014; Kim et al., 2017; Bhalla et al., 2018), and an improved kraft pulping efficiency as compared to wild type (Zhou et al., 2017). Monolignol *p*-coumarate esters have also been engineered in poplar, via expression of a rice *p-COUMAROYL-CoA:MONOLIGNOL TRANSFERASE* (*PMT*) gene, resulting in a higher frequency of resistant interunit bonds and a higher frequency of G and S terminal units with free phenolic groups (Smith et al., 2015; Sibout et al., 2016). While in Arabidopsis the heterologous expression of *PMT* resulted in a reduced lignin amount accompanied by an increased saccharification efficiency (Sibout et al., 2016), there was no decrease in lignin amount in poplar and the saccharification efficiency was not determined (Smith et al., 2015).

While several modifications of the lignin amount and composition were shown to provide improvements in biomass processing, these modifications were often accompanied by a biomass yield penalty (Leplé et al., 2007; Wadenbäck et al., 2008; Wagner et al., 2009; Voelker et al., 2010; Stout et al., 2014; Van Acker et al., 2014; Sykes et al., 2015; Zhou et al., 2018). A recent metastudy perturbed 21 lignin biosynthesis genes in *P. trichocarpa*, and comprehensively integrated the results of transcriptomic, proteomic, fluxomic, and phenomic data of 221 lines. The authors concluded that tree growth is not associated with lignin amount, subunit composition or specific linkages (Wang et al., 2018), but rather correlated with the presence of collapsed xylem vessels (Coleman et al., 2008a,b; Wagner et al., 2009; Voelker et al., 2010; Vargas et al., 2016; De Meester et al., 2018), the activation of a cell wall integrity pathway (Bonawitz et al., 2014) and/or the accumulation of chemical inhibitors (Gallego-Giraldo et al., 2011; Muro-Villanueva et al., 2019).

Whereas substantial efforts have been made to decrease lignin content by downregulation of lignin biosynthetic genes, studies on the upregulation of the lignin pathway and the overproduction of lignin have been scarce. Indeed, reports on the overexpression of *F5H* show an unchanged or even a decrease in lignin content (Huntley et al., 2003; Li L. et al., 2003; Stewart et al., 2009; Mansfield et al., 2012; Edmunds et al., 2017). The overexpression of *CAD* and *COMT* has resulted in gene-silencing rather than upregulation, or no effect on expression levels was detected (Baucher et al., 1996; Lapierre et al., 1999; Jouanin et al., 2000; Leplé et al., 2007; Van Acker et al., 2014). The overexpression of the R2R3-MYB transcription factors *PtoMYB92, PtoMYB216*, and *PtoMYB74* all resulted in additional xylem layers, thicker xylem cell walls as well as ectopic lignin deposition, and the plants accumulated 13–50% more lignin (Tian Q. et al., 2013; Li C.F. et al., 2015; Li et al., 2018). The *MYB* overexpression lines constitutively upregulated the lignin biosynthesis pathway genes, and while plants overexpressing *MYB92* and *MYB74* had a biomass penalty, the overexpression of *MYB216* resulted in plants with up to 50% more lignin and no developmental phenotype. As lignin is increasingly being considered an important resource for the sustainable production of chemicals (Cao et al., 2018) the engineering of plants overproducing lignin should be further explored.

## Field Trials

The examples discussed above clearly show that lignin engineering via down- or upregulation of phenylpropanoid pathway genes – or expression of heterologous genes – has the potential to increase the processing efficiency of lignocellulosic biomass. Due to practical and regulatory reasons, most studies report on data obtained from the analysis of trees grown in a greenhouse. However, experiments with trees grown in a greenhouse typically do not take into account developmental processes such as growth cessation and dormancy. In addition, greenhouse experiments do not provide sufficient insight into the interaction of the engineered plant with environmental factors such as soil type, wind, and pathogens. Understanding these interactions is an important step in the translation of research results toward commercial applications. Indeed, the body of work produced by studies for which permission to establish field trials was granted, highlights important differences in phenotype between greenhouse- and field grown trees. Table 1 summarizes the reports on field trials performed with *4CL, CCoAOMT, CCR, COMT*, and *CAD* downregulated trees.

Confirming the potential of modified lignocellulosic biomass as a substrate for applications, several lignin-engineered field-trial grown trees showed improvements in wood processing. Poplars downregulated for *CCoAOMT* grown for 5 years in a field trial in Beijing (China), showed an increased glucose and xylose release upon saccharification (Wang et al., 2012). Poplars downregulated for *CCR* and grown in a field trial in France, proved to be more amenable to chemical kraft pulping (Leplé et al., 2007). Two additional field trials conducted in France and Belgium with *CCR-*downregulated poplars resulted in up to 160% improvement in ethanol production in simultaneous saccharification and fermentation (SSF) assays; however the plants had up to 50% biomass reduction (Van Acker et al., 2014). Field trials with *CAD-*downregulated poplar also showed promising results. These trees showed slightly less lignin than wild type and proved more amenable to kraft delignification (Lapierre et al., 1999). Consistently, the same lines grown in larger-scale field trials in France and the United Kingdom showed a mild decrease in lignin amount and an improvement in kraft pulping deemed commercially relevant, since the plants needed 6% less alkali to achieve a delignification similar to that of wild-type trees (Pilate et al., 2002).

However, conflicting reports on both biomass yield and downstream processing efficiency suggest that these parameters are highly influenced by environmental factors. While a field trial conducted in China using *4CL* downregulated poplars found that, even with a 28% decrease in lignin content compared to wild type, the trees had about 8% increased height (Tian X.M. et al., 2013), consistent with greenhouse studies (Hu et al., 1999), other field trials found that *4CL*-downregulated poplars had decreased biomass and were sometimes even dwarfed (Voelker et al., 2010; Stout et al., 2014; Marchin et al., 2017). Reports also diverge regarding downstream processing efficiencies of wood derived from these *4CL*-downregulated field-grown poplars. While up to 100% increase in sugar recovery was found for *4CL1-*downregulated trees (35S-driven antisense *4CL* construct) grown in a mountain site in the United States (Xiang et al., 2017), data obtained from field studies conducted in Oregon (United States) found that *Pt4CL1* promoter-driven antisense silenced *4CL1* poplars had no improvement in saccharification efficiency compared to wild type (Voelker et al., 2010). Likewise, a long term study in Wenling (China), found that *4CL*-downregulated poplars did not show a significant improvement in sugar yield compared to wild type (Wang et al., 2012). In both latter cases, the trees showed mild decreases in lignin amount which did not translate into higher processing efficiency, potentially because of the higher concentration of extractives that could interfere with enzymatic activity (Voelker et al., 2010).

Field trial studies have shown that environmental factors can influence lignification and restore traits to wild-type levels as compared to the levels achieved when the same plants were grown in the greenhouse. While *4CL-*downregulated trees had decreased lignin content when grown under greenhouse conditions, analysis of the same *4CL* antisense poplars, but grown in the field, has often shown that the lignin content was increased as compared to the greenhouse-grown trees and sometimes even restored to wild-type levels (Stout et al., 2014; Xiang et al., 2017). Similarly, lignin levels were much less reduced in CCR-deficient poplars when they were grown in the field as compared to when they were grown in the greenhouse (Van Acker et al., 2014). At least for the CCR-deficient poplars, it is possible that the higher lignification level of field-grown trees is due to the fact that the wood samples were taken during winter. When tree growth ceases in autumn, the trees still have time to fully lignify their cell walls by the time the tree enters dormancy, as compared to greenhouse-grown trees that develop new xylem continuously. Lignin composition has also been shown to differ between greenhouse- and field-grown low-lignin trees. *4CL-*downregulated poplars grown in a field in North Carolina had lignin with a lower S/G than when the same lines were grown in the greenhouse (Stout et al., 2014).

Taken together, these results show that data obtained from greenhouse-grown trees cannot easily be extrapolated to field-grown trees, underpinning the need for field trial experiments at different locations. Some lines presented a yield penalty rendering them less interesting for applications, highlighting the need for a better understanding of the molecular basis of the yield penalty and the development of strategies to overcome this problem.

Lignin has been shown to play an important role in pathogen resistance (Miedes et al., 2014; Zhao and Dixon, 2014), and it plays a pivotal role in allowing the plant to transport water. This suggests that lignin modifications could have an impact on plant stress tolerance. While further investigation is needed to fully address this possibility, the downregulation of *4CL*, *COMT*, and *CAD* in poplar did not dramatically alter the feeding performance of leaf-feeding herbivores (Tiimonen et al., 2005; Brodeur-Campbell et al., 2006; Hjalten et al., 2013). The effect of the downregulation of *COMT* and *CAD* in poplar on plant-insect interactions has also been assessed on field-grown trees, and it was shown that the lignin-modified trees had normal incidence of visiting and feeding insects, as well as normal responses to microbial pathogens (Pilate et al., 2002; Halpin et al., 2007). These results indicate that trees with modified lignin do not necessarily suffer more than wild-type plants from pests and diseases. Nevertheless, profiling of the endosphere bacterial microbiome of wood harvested from field-grown, *CCR*-downregulated poplars demonstrated shifts in the bacterial community, presumably because of the altered abundance of particular phenolic metabolites in the xylem (Beckers et al., 2017).

Considering the role of lignin in xylem function and structure, the water relations of a few low lignin-modified poplars have been assessed. *4CL*-downregulated poplars were found to have reduced hydraulic conductivity, potentially interfering with plant growth (Marchin et al., 2017). Hydraulic stress experiments with poplars downregulated for *CCR*, *COMT* or *CAD* showed that these plants had a lower resistance to cavitation, while maintaining normal xylem hydraulic conductivity and water transport (Awad et al., 2012). These results suggest that the growth of low-lignin mutants might be influenced by water availability. As for any new hybrid obtained from classical breeding, field tests are needed to evaluate field performance and stress tolerance of lignin-engineered trees.

## Prospects for Lignin Engineering in Forest Trees

**FIGURE 2:**
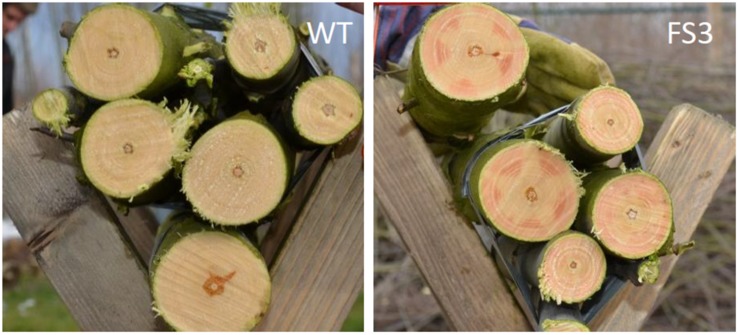
Patchy gene downregulation by RNAi. Patchy red xylem phenotype observed on trunks of CCR-deficient poplars **(right)** grown in a field trial in Belgium. The red xylem indicates areas of CCR downregulation. Wood from wild-type trees is whitish **(left)**.

The performance of lignin-engineered plants appears to be highly influenced by the environmental conditions. It is unclear, however, whether the differences observed between greenhouse-grown and field-grown trees, or between trees grown in different field locations, result from different levels of gene suppression or from interaction of the engineered trait with the environment (GxE). Indeed, unstable downregulation is a shortcoming of gene silencing techniques that are based on RNAi. This is witnessed by observing variation in the red xylem phenotype that is observed when particular lignin biosynthesis genes, such as *CAD*, *COMT*, or *CCR*, are downregulated. The red xylem coloration is often not uniform throughout the xylem, but rather appears in patches that reflect variable levels of gene silencing (Leplé et al., 2007; Voelker et al., 2010; Van Acker et al., 2014; Figure 2). In addition, the use of gene silencing methods can potentially result in concomitant silencing of closely related gene family members – perhaps to various degrees – clouding the interpretations and camouflaging the effects of downregulation of individual genes.

These issues can now be easily overcome by the use of CRISPR-based gene editing technologies that enable stable loss-of-function mutations (knock-outs) in specific target genes, allowing the dissection of the function of individual genes within families. For example, the targeting of individual *4CL* gene family members in poplar showed that 4CL1 is related to lignification, whereas 4CL2 is involved in proanthocyanidin production (Zhou et al., 2015). In addition to knock-out alleles, CRISPR-based gene editing also allows to create new alleles that confer partial reduction in enzyme activity. This opens the possibility to fine-tune the level of residual enzyme activity and to bypass the yield penalty that is often observed when lignin amount drops below a threshold level. Another promising avenue for lignin engineering in forest trees made possible through CRISPR-based genome engineering is the simultaneous editing of multiple genes (allele stacking) to optimize biomass processing efficiency, as exemplified in Arabidopsis where stacking of the *transaldolase* (*tra)* and *comt* mutations, the *c4h* and *comt* mutations, or the *4cl* and *comt* mutations resulted in additive and synergistic improvements in saccharification efficiency (de Vries et al., 2018). Indeed, a systems approach in *P. trichocarpa* predicts that the concomitant downregulation of *PAL* and *CCoAOMT*, or *PAL*, *C3’H* and *CCOAOMT* will substantially improve wood properties and sugar release (Wang et al., 2018).

**FIGURE 3:**
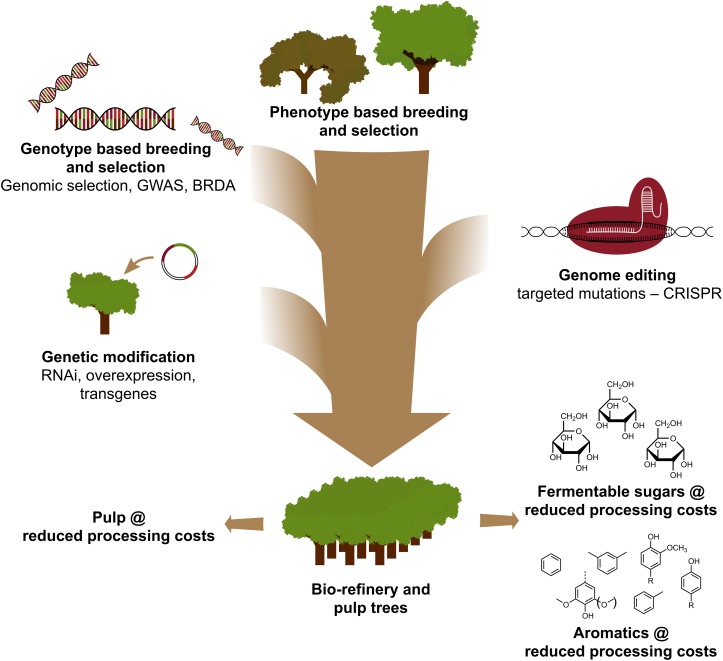
Genetic improvement of forest trees through a combination of breeding tools. To accelerate the genetic improvement of forest trees for pulp and biorefinery applications, classical and new breeding tools need to be smartly combined. Classical breeding involves phenotypic selection of trees for controlled crosses, followed by phenotypic selection. With the advent of genome sequence information of many forest trees, new strategies such as Genomic Selection, Genome Wide Association Studies (GWAS) and Breeding with Rare Defective Alleles (BRDA) have been developed to speed up the capture and enrichment of DNA polymorphisms associated with beneficial traits. CRISPR-based genome editing allows to modify the genome in a way that mimics natural polymorphisms. Genetic modification involves the stable integration of foreign DNA into the tree to overproduce (an) enzyme(s) or downregulate (a) gene(s). Combining the classical and New Breeding Techniques is needed to provide sufficient highly quality wood for society.

The use of CRISPR-based genome editing in tree improvement for the pulp and paper and the bio-refinery industries, as well as for the production of platform aromatics from the hydrogenolytic breakdown of lignin, will be most valuable when this technology is strategically combined with other breeding techniques (Figure 3). Indeed, large variation in lignin amount and S/G composition already exists in natural populations of forest trees (Studer et al., 2011). Given that both traits affect the glucose release upon saccharification (Yoo et al., 2018), exploiting this genetic diversity by conventional breeding, aided by Genome Wide Association Studies (GWAS) (Porth et al., 2013; Fahrenkrog et al., 2017; Liu et al., 2018), Breeding with Rare Defective Alleles (BRDA) (Vanholme et al., 2013a) or genomic selection (Yin et al., 2010; Muchero et al., 2015; Pawar et al., 2018; Xie et al., 2018), is a valuable strategy to obtain lines that have improved wood processing efficiency. Once elite trees are obtained by these breeding methods, genetic engineering and CRISPR-based gene editing of specific genes is a very promising avenue to further improve these elite genotypes without breaking up their genetic constitution and without going through lengthy breeding cycles. Given the imminent climate crash, we have no more time to lose in adopting these new breeding techniques in our race to the biobased economy.

## Author Contributions

All authors listed have made a substantial, direct and intellectual contribution to the work, and approved it for publication.

## Conflict of Interest Statement

The authors declare that the research was conducted in the absence of any commercial or financial relationships that could be construed as a potential conflict of interest.
